# Anesthetic management of a patient with narcolepsy by combination of total intravenous and regional anesthesia: a case report

**DOI:** 10.1186/s40981-017-0107-4

**Published:** 2017-07-14

**Authors:** Daiki Takekawa, Tetsuya Kushikata, Masato Kitayama, Kazuyoshi Hirota

**Affiliations:** 1 Department of Anesthesiology, Hirosaki University Graduate School of Medicine, 5 Zaifu-cho, Hirosaki, 036-8562 Japan; 2 Division of Surgical Center, Hirosaki University Hospital, 53 Hon-cho, Hirosaki, 036-8563 Japan

**Keywords:** Narcolepsy, Orexin, General anesthesia

## Abstract

Narcolepsy is a neurological disease characterized by excessive daytime sleepiness, cataplexy, and/or a sudden loss of muscle tone due to malfunction of the orexinergic system, which may cause delayed emergence from general anesthesia. We report a successful anesthetic management of 24-year-old female narcoleptic patient undergoing left anterior cruciate ligament reconstruction. Anesthesia was induced and maintained with total intravenous anesthesia (TIVA) using propofol and remifentanil. Ultrasound-guided left femoral nerve block was also performed with 0.375% ropivacaine 20 ml. Acetaminophen 1000 mg was intravenously administered as part of a multimodal analgesia. After the surgery, the trachea was extubated 9 min after termination of TIVA, and then, the patient correctly responded to verbal commands. The postoperative course was uneventful without any narcoleptic symptoms.

## Background

Narcolepsy is a neurological disease characterized by excessive sleep during the day, catalepsy, and sleep paralysis due to malfunction of the orexinergic (oxergic) system such as loss of OXergic neurons and deficiency of orexins (Oxs) [1]. OXergic neurons widely project throughout the central nervous system (CNS) such as the noradrenergic locus coeruleus, the cholinergic basal forebrain, the dopaminergic ventral tegmental area, the serotonergic raphe nuclei, and the histaminergic tuberomammillary nucleus to regulates various physiological functions including not only sleep wakefulness but also analgesia, sympathetic nervous system, feeding behavior, and emotional behavior [1, 2].

The mechanism of loss of consciousness by general anesthesia partially includes the activation and suppression of endogenous sleep- and wakefulness-promoting pathways [3]. Therefore, it is possible that activation of orexinergic nervous system decrease anesthesia times. In fact, it was reported that intracerebroventricular administration of orexin significantly decreased general anesthesia time in the rat [4–6]. Thus, prolonged emergence from general anesthesia would be expected in patients with narcolepsy. The desirable anesthetic management of patients with narcolepsy is to decrease the dosage of general anesthetic and analgesic agents and/or use short-acting agents to prevent delayed emergence. Thus, the combination of general and regional anesthesia may be one of the best options.

Here, we report anesthetic management of a narcoleptic patient undergoing left anterior cruciate ligament (ACL) reconstruction under a combination of total intravenous anesthesia (TIVA) with femoral nerve block.

## Case presentation

We have obtained a written informed consent from the patient and healthy volunteers for publication of this case report and measurement of plasma OXA concentrations before and 1 h after anesthesia induction and at emergence from anesthesia using a commercial enzyme-linked immunosorbent assay kit (Peninsula Laboratories, San Carlos, CA).

A 24-year-old woman (160 cm, 61 kg) with narcolepsy was scheduled to undergo left ACL reconstruction. The patient took prescribed modafinil 200 mg to treat excessive sleepiness with activation of histaminergic and OXergic neurons in narcoleptic patients [7]. However, she sometimes claimed intolerable daytime drowsiness. She did not have any other abnormal medical history and abnormal physical examination data.

On the morning of the surgery, she took her daily dose of modafinil and roxatidine 75 mg as anesthetic premedication but other routine premedications such as benzodiazepines were avoided not to be sedated deeply. Indeed, the BIS was over 90 before induction of general anesthesia. Anesthesia was uncomplicatedly induced by propofol 120 mg, remifentanil 0.5 μg/kg/min, and rocuronium bromide 40 mg followed by tracheal intubation. After the induction, ultrasound-guided left femoral nerve block was performed with 0.375% ropivacaine 20 ml. Anesthesia was maintained with propofol 6–8 mg/kg/h and remifentanil 0.1–0.2 μg/kg/min to keep BIS value between 40 and 60. Intravenous acetaminophen 1000 mg was also administered as a part of multimodal analgesia. As hemodynamics were stable during anesthesia, vasoactive agents were not required. The duration of surgery was 50 min. The patient emerged from anesthesia and extubated 9 min after discontinuation of propofol and remifentanil infusion. Her consciousness was clear and the BIS values were above 90. Intravenous fentanyl 100 μg was required for relief of pain of sciatic nerve region.

She moved to the intensive care unit (ICU) for postoperative care. The postoperative course was uneventful without any hemodynamic instability, respiratory depression, and progress of narcoleptic symptoms. Then, she was discharged on the 9th postoperative day.

The measured plasma OXA were always lower than the data of healthy adult volunteers (Table 1).

**Table 1:** Perioperative changes in plasma orexin A concentrations in a narcoleptic patient

	Patient’s data		
Normal range (mean ± SD)	Before anesthesia	1 h after anesthesia	Emergence
0.277 ± 0.115 ng/ml	0.085 ng/ml	0.119 ng/ml	0.153 ng/ml

Normal range: Average of plasma OXA concentrations of four healthy volunteers in our department which was measured with the patient’s plasma

### Discussion

Anesthetic management of patients with narcolepsy has yet to be established, because of the rarity of the disease. It is considered that effects of anesthetics depend on the severity of the disease. Cavalcante and colleagues [8] recently reported a case-control study for determination of the perioperative risk of narcoleptic patients undergoing general anesthesia compared with matched control patients (*n* = 76 each). They found that narcoleptic patients compared to control patients were more often prescribed CNS stimulants (73.7 vs 4.0%, *P* < 0.001) and antidepressants (46.1 vs 27.6%, *P* = 0.007) and more often revealed obstructive sleep apnea (40.8 vs 19.1%, *P* < 0.001) in the preoperative period. Although intraoperative course was similar because of no differences in the using frequency of vasoactive agents and fluid administration between groups, narcoleptic patients had a higher frequency of emergency response team (ERT) activations (6.6 vs 1.3%, *P* = 0.04) in postoperative period. ERT activation was caused by hemodynamic instability such as hypotension, tachycardia, or bradycardia in all patients. One narcoleptic patient showed excessive sedation with respiratory depression. Thus, narcoleptic patients may have higher postoperative risk.

As our patient claimed intolerable daytime drowsiness, the narcoleptic symptoms were not sufficiently controlled by modafinil. Therefore, we gave a daily dose of modafinil that would improve anesthesia recovery [9], avoided sedative premedication, and chose propofol-remifentanil TIVA with femoral nerve block under BIS monitoring. Propofol and remifentanil are well-known to be short-acting agents to avoid residual effects. BIS monitoring was also useful to titrate anesthetic agents [10] and detect narcolepsy-catalepsy episode [11]. In addition, to reduce opioid, which may cause delayed emergence, for analgesia, multimodal analgesia such as femoral nerve block and iv acetaminophen was performed. Indeed, the patient was emerged from general anesthesia within 10 min without narcoleptic symptoms following discontinuing propofol. The dose of propofol and remifentanil in this patient was quite normal compared to non-narcoleptic patients.

Fentanyl was required for postoperative analgesia in this patient. Although in our hospital to avoid the delay of operation schedule for knee surgeries, we do not perform sciatic nerve block, we should have done it in this patient to avoid postoperative narcotic analgesia.

We found that OXA level of this patient is lower compared with control values obtained from some healthy human. Plasma OXA concentrations in this patient were lower than that of awake healthy adult volunteers and patients under general anesthesia [12]. Although it is clinically difficult to determine whether plasma OXA originates from the central nervous system, Higuchi and colleagues reported that plasma OXA concentration in narcolepsy patients was lower than that in healthy volunteers [13]. In addition, plasma OXA in this patient increased at emergence from TIVA. This increase was similar to the data of our previous report [12]. Thus, the degeneration of OXergic neurons might yet to be completed although we did not measure the cerebrospinal fluid concentration of OXA.

## Conclusions

In summary, we could successfully manage an anesthetic case of a narcoleptic patient with a combination of TIVA and regional anesthesia.
